# Developing and Validating an Item Bank for Alcohol Use Disorder Screening in the Chinese Population by Using the Computerized Adaptive Testing

**DOI:** 10.3389/fpsyg.2020.01652

**Published:** 2020-07-08

**Authors:** Jie Lian, Yan Cai, Dongbo Tu, Chongqin Xi

**Affiliations:** School of Psychology, Jiangxi Normal University, Nanchang, China

**Keywords:** alcohol use disorder, item bank, item response theory, computerized adaptive testing, psychometric measurement

## Abstract

**Objective:**

To detect the individual’s severity of alcohol use disorder (AUD) in an effective and accurate manner, this study aimed to build an item bank for AUD screening and derive the computerized adaptive testing (CAT) version of AUD (CAT-AUD).

**Methods:**

The initial CAT-AUD item bank was selected from the Chinese version of the questionnaires related to AUD according to the DSM-5 criteria. Then 915 valid Chinese samples, covering the healthy individuals and the AUD high-risk individuals, completed the initial CAT-AUD item bank. By testing the unidimensionality, test fit, item fit, discrimination parameter and differential item functioning of the initial item bank, the final CAT-AUD item bank confirming to the requirements of the item response theory (IRT) were obtained. Subsequently, the CAT-AUD simulation study based on the real data of the final item bank conducted to detect characteristics, reliability, validity, and predictive utility (sensitivity and specificity) of CAT-AUD.

**Results:**

The CAT-AUD item bank meeting the IRT psychometric measurement requirements could be well geared into the graded response model. The Pearson’s correlation between the estimated theta via CAT-AUD and the estimated theta via the full-length item bank reached 0.95, and the criterion-related validity was 0.63. CAT-AUD can provide highly reliable test results for subjects whose theta above −0.8 with an average of 16 items. Besides, the predictive utility of CAT-AUD was better than AUDIT and AUDIT-C.

**Conclusion:**

In brief, the CAT-AUD developed in this study can effectively screen the AUD high-risk group and accurately measure the AUD severity of individuals.

## Introduction

As one of the world’s commonest mental disorders, alcohol use disorder (AUD) is a problematic pattern of alcohol use which could lead to clinically significant impairment or distress (American Psychiatric Association, 2013). In 2016, the harmful drinking pattern had resulted in an estimated 3 million deaths worldwide, which accounted for 5.3% of all deaths (World Health Organization, 2019). This disorder is also correlated with a wide range of physical and mental disorders (Cargiulo, 2007; Hasin et al., 2007; Rehm, 2011). Individuals with AUD are not competent in fulfilling their primary role at the workplace, school or home, while their social relationship and the financial situation will be adversely affected (Rehm et al., 2009; American Psychiatric Association, 2013). Besides, AUD high-risk individuals are inclined to cause social harm such as vehicle accidents and violent crime (World Health Organization, 2014).

In China, public health has also been endangered by AUD. Surveys of several provinces and cities showed that AUD had jumped to the forefront among mental diseases and became a serious public health problem in China (Shi et al., 2005; Pan et al., 2006; Li et al., 2008; Ruan et al., 2010). To be specific, there are some differences in the probability of suffering AUD among different demographic groups. In terms of gender, Zhang et al. (2010) found that the rate of suffering AUD in adults was 10.92% for males and 0.17% for females in Shandong Province. The study by Chang et al. (2016) also showed a statistically significant difference in suffering AUD among genders (χ^2^ = 125.598, *p* < 0.01). In urban and rural areas, the survey results of adults from Heilongjiang Province showed that the harmful drinking rate of the urban population was 5.20%, and that of the rural population was 9.63%, which was higher in rural areas than in urban areas (χ^2^ = 22.27, *p* < 0.05).

For the purpose of reducing the rate of prevalence and mortality caused by AUD, detections should be used in the general population to screen out the AUD high-risk group. Currently, numerous questionnaires such as the Alcohol Use Disorder Identification Test (AUDIT; Babor and Grant, 1989), the classical Michigan Alcoholism Screening Test (MAST; Selzer, 1975) and the Alcohol Dependence Scale (Skinner, 1981) have been developed to measure the specific symptoms rather than the whole symptoms of AUD under the framework of classical test theory (CTT). Nevertheless, several inherent defects were showed in these questionnaires. First, current questionnaires were not developed in accordance with the fifth edition of the Diagnostic and Statistical Manual of Mental Disorders (DSM–5; American Psychiatric Association, 2013) criteria. Thus, some symptoms of AUD in DSM-5 could not be mirrored integrally by the existing questionnaires which were not keeping abreast with times and practice, and then the information offered by questionnaires would remain unreliable. Second, based on the principle of CTT, participants would respond to the same items which are equally weighted regardless of their level of some trait, and the summed total scores under the normal condition were the measurement results which would be representative of individuals’ level of some trait. As a matter of fact, each item has a different weight for AUD high-risk individuals of different levels of severity. Some researchers reported that AUD is a heterogeneous disorder and upheld for personalized treatment of AUD (Linden-Carmichael et al., 2018). Also, Weiss (2004) stated that some items in fixed-length instruments may lead to error since measurement precision declined at both the high and low levels of the trait. On the whole, these CTT based questionnaires for AUD are not comprehensive or adaptive sufficiently and should be required further improvement.

Traditional CTT had become inadequate for the demand for measurement accuracy and efficiency. Under the stimulation of such need, item response theory (IRT; Embretson and Reise, 2013) was developed. The characteristic of IRT is to describe in the form of probability function how the item response result is affected by the combination of the ability level of the subject and the item characteristic, which entitles IRT with many advantages. First, it has parametric invariance, that is, the population parameter estimated from different samples of the same population is constant. Secondly, the subjects’ parameter estimated by IRT and the measuring scale of the item difficulty parameter is unified (Baker, 2001). In this way, items whose difficulty parameter is suitable for the subjects’ ability level can be selected to make the estimates of individuals’ latent trait more efficient and accurate. Furthermore, IRT can accurately estimate the measurement error of each item and the total test for subjects with different trait levels (Kim, 2012) while the traditional CTT which calculated the standard error of measurement (SEM) for a group of scores could just offer the total reliability regardless of subjects’ trait levels. In recent years, CTT was applied to conditional standard error of measurement (CSEM), which could also make CTT provide high measurement reliability for groups with different ability levels (Culpepper, 2013).

The emergence and growth of computerized adaptive testing (CAT) were due to the creation of IRT and the inexpensive accessibility to high-speed desktop computers (Gershon, 2005). The core of CAT is to maximize the precision by only administering the items suitable to individuals’ latent trait (van der Linden and Glas, 2000, van der Linden and Glas, 2010; Wainer et al., 2000). In accordance with IRT (Embretson and Reise, 2013), the computer flexibly administers suitable items that have been calibrated for individuals by specific algorithms, so people are allowed to administer fewer items when the accuracy is ensured to be as much as possible (Hambleton et al., 1992; van der Linden and Hambleton, 1997). The adaptive algorithm guarantees the test accuracy of the subjects and makes the subjects with different latent traits answer questions with different parameters as few as possible. With such an adaptive ability in CAT, the shortcomings of current questionnaires can be compensated. To accurately measure an individual’s level of AUD, selecting the CAT version may be prioritized.

This study aimed to develop a CAT version of AUD (CAT-AUD) based on DSM-5 as a screening instrument to distinguish the healthy group and the AUD high-risk group in Chinese adult population, which is expected to be a comprehensive, effective, and highly accurate for the AUD assessment. Based on the guidance of the AUD criteria of DSM-5, items that measure at least one symptom of AUD in DSM-5 were selected from five famous AUD scales to constructed the initial CAT-AUD item bank. After that, various statistical analyses including unidimensionality test, monotonicity test, local independence test, the level of item-fit test, the item discrimination and differential item function (DIF) test based on IRT were conducted to the initial item bank to develop the final item bank. Finally, a CAT-AUD simulation was implemented to investigate the reliability, validity and prediction utility (sensitivity and specificity) of the CAT-AUD.

## Materials and Methods

### Participants

One thousand three hundred and twenty Chinese participants aged beyond 18 years from Henan, Jiangxi, Shandong, Hunan, Guangxi, Fujian Provinces were recruited for this survey. Before conducting the questionnaire, each participant was informed of the individual privacy protection principle and then they volunteered to participate in the survey. The informed consent of all participants was obtained in accordance with the declaration of Helsinki. The survey included the basic demographic information (gender and region), the AUD item bank (including three pairs of lie detection items), and questions used as the exclusion criteria. In order to obtain real and effective response data, we screened the original questionnaires in advance. If participants satisfy one of the exclusion criteria below, they would not be covered in this study: (1) those patients with psychosis illness; (2) those having received psychiatric medication in a week; (3) those abusing or relying on other substances other than tobacco; (4) those with impaired heart, liver and kidney function or other serious physical diseases not resulting from alcohol. Besides, if a participant had at least one pair of identical response data on the lie detection item, his or her response data would be excluded.

One thousand three hundred and twenty questionnaires were distributed and 1128 completed questionnaires were collected, with a recovery rate of 85.5%. Among them, 52 questionnaires contained missing demographic information, 73 questionnaires failed to meet the lie detection test criteria, 88 questionnaires met at least one of the exclusion criteria, then the number of valid questionnaires was 915, suggesting that the valid rate was 81.1%. Table 1 contains the demographic and clinical characteristics of the valid sample. Among these participants, 735 were males (80.33%), with the average age of 37.58 (*SD* = 14.28), and 180 were females (19.67%), with the average age of 33.95 (*SD* = 11.38). There were 420 subjects in urban areas (45.90%), aged 35.83 on average (*SD* = 11.40), and 495 subjects in rural areas (54.10%), aged 36.21 on average (*SD* = 11.51).

**TABLE 1:** Demographic and clinical characteristics of the sample (*N* = 915).

**Variable**	**Category**	**Frequency**	**Percent (%)**
Gender	Male	735	80.33
	Female	180	19.67
Age	Under 30	295	32.24
	30 and above	546	59.67
	Missing	74	8.09
Region	Urban areas	420	45.90
	Rural areas	495	54.10
Clinical information	Healthy	578	63.17
	High-risk AUD	337	36.83

All participants took part in the survey above as well as took part in the questionnaire of the MAST. In accordance with the research of Selzer (1975), subjects with a total score of 6 or above were classified as the AUD high-risk group, while those with a score of <6 were classified as the healthy group. These two groups were used to test the validity of the CAT-AUD which would be constructed subsequently.

### Instruments

The authors searched domestic and foreign publications with words such as the Chinese version scale of “AUD,” “AUD scale,” and “AUD questionnaire.” In order to find the target questionnaires among the numerous questionnaires, we selected the questionnaires related to the AUD according to some certain criteria: (1) whether there is a validated Chinese version of the questionnaire; (2) whether the items in the questionnaire measure one of the AUD symptoms in DSM-5; (3) whether the reliability and validity are high enough: (a) the test-retest reliability or the Cronbach’s alpha of a questionnaire is higher than 0.8 (Wu, 2010); (b) the criterion-related validity of the questionnaire is higher than 0.4 (Streiner and Norman, 2008); (c) the scale content validity (S-CVI) is higher than 0.8 (Davis, 1992); (4) the high citation frequency of a questionnaire; (5) the number of items of a questionnaire should not be too small. For the original questionnaire without a Chinese version, the process of Chineseization is as follows: after obtaining the approval of the author of the original questionnaire, the Chinese version of the questionnaire was then translated using the Brislin’s classical back translation model to form the Chinese version of the questionnaire. Moreover, to compare the reliability and validity of different questionnaires for the sample in this study horizontally, the Crombach’s alpha and criterion-validity values of the Chinese version of questionnaires above were calculated and displayed.

The Chinese version of AUD scales employed in this study exhibited three major purposes. Five scales including the Alcohol Dependence Scale (ADS; Skinner, 1981), the Munich Alcoholism Test (MALT; Speckens et al., 1991), the Severity Alcohol Dependence Questionnaire (SADQ; Stockwell et al., 1983; Cheng et al., 2009), the Obsessive Compulsive Drinking Scale (OCDS; Anton et al., 1995), and the Approach and Avoidance of Alcohol Questionnaire (AAAQ; McEvoy et al., 2004) were used to build a comprehensive CAT-AUD item bank containing all AUD symptoms of DSM-5; the classical Michigan Alcoholism Screening Test (MAST; Selzer, 1975; Hsueh et al., 2014) was performed as a criterion scale to investigate the validity of the CAT-AUD; the Alcohol Use Disorder Identification Test (AUDIT; Babor and Grant, 1989; Zhang et al., 2017) and the AUDIT consumption questions (AUDIT-C; Bush et al., 1998) were applied for the comparison of the performance of the CAT-AUD.

#### The Alcohol Dependence Scale

The ADS was developed using the alcohol dependence factor correlated with chronic social debilitation and psychopathology (Skinner, 1981) to ascertain patients’ symptoms of alcohol. The ADS covered 29 items scored with 0–1, 0–2, and 0–3, respectively. The original ADS scale was used, which exhibited the internal consistency reliability of 0.92 and the criterion-related validity of 0.69 with MAST (Skinner and Allen, 1982) and the mean and standard deviation (SD) of the sample ADS score were 23.1 and 11.3, respectively. In the present study, the Chinese version of the ADS depicted a Cronbach’s alpha of 0.85 and the criterion-related validity of 0.69 with MAST.

#### The Munich Alcoholism Test

The MALT refers to a screening instrument for alcoholism especially applicable to medical populations (Speckens et al., 1991). Such a scale is commonly used in many counties worldwide. This study chose the self-rating part of MALT (MALT-S) with 24 dichotomously scoring items. The scale exhibited split-half reliability of 0.94 and convergent validity of 0.85 (Feuerlein et al., 1979). In the present study, the Chinese version of the MALT depicted a Cronbach’s alpha of 0.86 and the criterion-related validity of 0.74 with MAST.

#### The Severity Alcohol Dependence Questionnaire

The SADQ covered 20 items all graded in four levels (Stockwell et al., 1983). The SADQ consisted of five dimensions: (a) physical symptoms of withdrawal (PHYS), (b) affective symptoms of withdrawal (AFF), (c) craving and withdrawal-relief drinking (NEED), (d) typical daily consumption (ALC), as well as (e) rapidity of reinstatement of symptoms after a period of abstinence (POSTAB) (Stockwell et al., 1979). Given the study of Stockwell et al. (1979), the questionnaire’s test-retest reliability was 0.85, and the criterion-related validity was 0.63. For the sample who rated 0 or 1, the mean and SD of the SADQ score were 28.8 and 11.3, respectively. For sample who rated 2, the mean and SD of the SADQ score were 50.6 and 12.2, respectively. In the present study, the Chinese version of the SADQ (Cheng et al., 2009) depicted a Cronbach’s alpha of 0.95 and the criterion-related validity of 0.61 with MAST.

#### The Obsessive Compulsive Drinking Scale

The OCDS, developed by Anton et al. (1995) who modified Modell’s Yale-Brown Obsessive Compulsive Scale (Y-BOCS), refers to a self-reported instrument that is capable of rating the level of drinking crave (Modell et al., 1992). It consists of 14 items, all of which were scored from 0 to 4. Among them, the item numbers of 1–6 acted as the degree of psychological craving for alcohol use testers, and the item numbers of 7–14 indicated the testers’ behavior and motivation for alcohol consumption. The test-retest reliability of the OCDS was 0.96, and the convergent validity was 0.83, which are both relatively high (Anton et al., 1995) when the mean and SD of the sample score were 22.5 and 7.5, respectively. In the present study, the Chinese version of the OCDS depicted a Cronbach’s alpha of 0.92 and the criterion-related validity of 0.68 with MAST.

#### The Approach and Avoidance of Alcohol Questionnaire

The AAAQ has been developed to assess inclinations to drink and not drink separately (McEvoy et al., 2004). Participants suggested that how firmly they agreed with each item on a nine-point Likert-type scale, from “not at all” (0) to “very strongly” (8). Existing studies suggested that the AAAQ had high internal consistency and convergent and predictive validity (McEvoy et al., 2004; Klein et al., 2007). According to McEvoy et al. (2004), the internal consistency of AAAQ was 0.62, and the scale interpreted a substantial proportion of the variance in drinking frequency (41–53%), drinking quantity (49–60%) as well as drinking problems (43%). For the inclined sample, the mean and SD of score were 4.16 and 2.42, respectively. For the obsessed sample, the mean and SD of score were 1.56 and 1.54, respectively. In the present study, the Chinese version of the AAAQ depicted a Cronbach’s alpha of 0.89 and the criterion-related validity of 0.46 with MAST.

#### The Michigan Alcoholism Screening Test

The MAST refers to a self-rating scale used in this study to screen high-risk individuals for alcohol disorders (Selzer, 1975). MAST primarily measures five aspects (namely, problems recognized by oneself or others, work and social problems, seeking help for drinking, marital and family problems, as well as physical health problems). Compared with the AUD symptoms of DSM-5, these aspects might be more general but almost consistent, so this scale was adopted as the criterion scale. In this study, the Chinese version of MAST (MAST-C) was employed to investigate the validity of the CAT-AUD (Hsueh et al., 2014). The test consisted of 24 items, and the item response options were rated dichotomously (yes/no). The internal consistency reliability and the scale-level content validity index (S-CVI) of the MAST-C were 0.89 and 0.92, respectively (Hsueh et al., 2014). In the present study, the Chinese version of the MAST (Hsueh et al., 2014) depicted a Cronbach’s alpha of 0.88.

#### The Alcohol Use Disorder Identification Test

For the validity comparison, the AUDIT and the AUDIT-C were employed in the study. The AUDIT was built by investigators from WHO collaborated on the six-nation project in the 1980s (Saunders, 1987; Babor and Grant, 1989; Babor et al., 1992; Saunders et al., 1993). The results of the study by Zhang et al. (2017) revealed that Cronbach’s alpha of the Chinese version was 0.782, and the split-half reliability was 0.711. The content validity of the item-level (I-CVI) ranged from 0.83 to 1.00, and the average content validity for the scale-level (S-CVI) was 0.99 (Zhang et al., 2017). In the present study, the Chinese version of the AUDIT (Zhang et al., 2017) depicted a Cronbach’s alpha of 0.90 and the Chinese version of the AUDIT-C depicted a Cronbach’s alpha of 0.77.

## Data Analyses

### The Initial Item Bank for the CAT-AUD

For subsequent analysis, items which measure at least one criterion of the DSM-5 for AUD for the constructive purpose were introduced into the initial item bank. In order to screen out items which met the above standard, we invited 3 clinicians with curing AUD experience to evaluate whether the items measure at least one criterion of the DSM-5 for AUD. By comparing the judgment results of different clinicians, items that measured at least one criterion were kept in the initial CAT-AUD item bank. Table 2 presents the source of the selected items in the item bank. The R package mirt (Version 1.29; Chalmers, 2012) was performed for the following IRT analyses.

**TABLE 2:** Item psychometric information of the final item bank (*n* = 113).

**Item code**	**Item parameters**				**Item-fit estimations**			**Item source**
	**Slope**	**Number of score categories**	**Smallest threshold**	**Highest threshold**	**S-χ^2^**	***df***	***p***	
25	1.000	2	–	0.548	131.984	106	0.044	ADS
26	1.508	2	–	1.536	86.887	81	0.307	ADS
27	1.579	2	–	1.807	76.067	68	0.235	ADS
29	1.502	2	–	1.659	82.305	79	0.377	ADS
30	0.989	2	–	1.226	119.155	110	0.259	ADS
31	1.500	2	–	1.839	94.872	69	0.021	MALT
32	0.931	2	–	1.177	122.807	112	0.228	MALT
33	1.467	2	–	1.695	84.795	78	0.280	MALT
34	1.314	2	–	1.411	117.281	99	0.101	MALT
35	1.504	2	–	1.157	108.433	99	0.243	MALT
36	1.634	2	–	1.159	103.482	95	0.259	MALT
37	1.553	2	–	1.192	95.974	97	0.510	MALT
38	1.635	2	–	1.318	77.861	87	0.748	MALT
39	1.237	2	–	1.166	123.590	106	0.117	MALT
40	1.303	2	–	1.431	100.283	99	0.445	MALT
41	0.857	2	–	0.898	104.824	112	0.672	MALT
44	1.431	2	–	1.067	116.514	103	0.171	MALT
45	0.817	2	–	1.422	105.849	113	0.671	MALT
46	0.758	2	–	0.263	116.939	106	0.220	MALT
47	1.591	2	–	1.353	58.336	86	0.990	MALT
48	1.394	3	1.022	2.730	108.312	106	0.419	ADS
49	1.657	3	1.347	3.077	92.744	85	0.265	ADS
50	2.238	3	1.089	2.337	89.713	83	0.288	ADS
51	1.408	3	0.643	1.887	110.598	94	0.116	ADS
52	2.025	3	1.507	2.298	79.635	69	0.179	ADS
53	2.025	3	1.331	2.462	109.234	79	0.014	ADS
54	1.839	3	1.138	1.937	81.096	94	0.826	ADS
55	2.883	4	1.222	2.823	95.494	68	0.016	SADQ
56	3.479	4	1.213	2.722	74.065	58	0.076	SADQ
57	3.873	4	1.204	2.595	80.043	55	0.015	SADQ
58	3.807	4	1.145	2.699	75.841	59	0.069	SADQ
59	3.873	4	1.176	2.642	67.676	57	0.157	SADQ
60	3.504	4	1.105	2.479	76.462	63	0.119	SADQ
61	3.365	4	1.205	2.661	64.740	63	0.416	SADQ
62	2.634	4	0.716	2.583	95.433	90	0.328	–
63	1.792	4	0.287	2.978	116.111	99	0.115	–
66	2.030	4	0.438	3.132	95.762	98	0.545	–
67	2.628	4	0.519	2.863	91.641	92	0.491	–
68	1.078	4	0.207	2.785	212.534	162	0.005	–
69	2.324	4	0.908	3.173	101.874	91	0.205	–
70	2.869	4	0.941	2.865	99.213	78	0.053	–
71	2.901	4	0.837	2.843	91.470	83	0.246	–
72	2.969	4	0.972	2.422	76.437	79	0.561	–
73	3.414	4	1.012	2.554	66.968	66	0.444	–
74	3.952	4	1.038	2.477	61.694	62	0.487	–
75	3.558	4	1.063	2.784	63.558	63	0.457	–
76	3.170	4	0.962	2.841	78.541	75	0.367	–
77	3.496	4	1.057	2.877	81.092	64	0.073	–
78	3.144	4	0.752	2.746	110.435	81	0.017	–
79	2.465	4	0.602	3.096	109.960	92	0.098	–
80	2.898	4	0.756	2.863	96.888	83	0.141	–
81	2.766	4	0.782	3.016	89.287	85	0.354	–
82	1.727	4	0.753	2.665	117.565	103	0.155	–
83	3.089	4	0.850	2.899	83.507	79	0.343	–
84	3.908	4	0.874	2.568	79.309	70	0.209	–
85	3.209	4	0.842	2.693	88.337	79	0.221	–
86	3.106	4	1.070	2.920	76.723	67	0.195	–
87	2.751	4	0.978	2.629	92.498	81	0.180	–
88	3.675	4	1.252	2.708	81.922	56	0.014	–
89	2.642	4	0.928	2.826	86.525	82	0.345	–
90	3.584	4	1.095	2.650	80.172	63	0.071	–
91	3.633	4	1.272	2.593	74.522	55	0.041	–
92	3.016	4	0.825	2.704	121.521	79	0.002	–
93	3.849	4	1.015	2.620	83.223	61	0.031	–
95	3.020	4	1.003	2.665	96.018	76	0.060	–
96	2.327	4	0.815	3.159	79.972	90	0.766	–
97	2.331	4	0.656	2.833	88.075	94	0.653	–
98	2.646	4	1.025	2.831	77.232	81	0.598	–
99	2.652	4	0.826	2.722	70.967	87	0.894	–
100	2.823	4	0.922	2.917	83.157	79	0.353	–
101	3.033	4	0.887	2.816	78.686	77	0.425	–
102	2.321	4	0.599	2.630	101.611	96	0.328	–
103	3.136	4	0.836	2.441	83.899	80	0.361	–
104	3.221	4	0.848	2.691	93.627	78	0.110	–
105	2.744	4	0.652	2.639	100.303	89	0.194	–
106	3.144	4	0.804	2.755	86.475	81	0.318	–
107	2.988	4	0.778	2.518	109.855	83	0.026	–
108	3.100	4	0.710	2.668	75.856	85	0.751	–
109	2.602	4	0.527	2.536	99.864	93	0.295	–
110	2.747	4	0.673	2.459	100.763	90	0.206	–
111	3.310	4	0.838	2.600	71.356	77	0.660	–
112	3.256	4	0.796	2.607	95.765	81	0.126	–
113	3.256	4	0.824	2.607	68.591	78	0.768	–
114	2.133	4	0.636	2.955	135.361	97	0.006	–
115	2.560	4	0.812	2.736	88.837	89	0.485	–
116	2.748	4	0.738	2.731	102.698	88	0.135	–
117	3.144	4	0.841	2.614	85.841	79	0.280	–
118	2.524	4	0.663	2.908	100.060	92	0.265	–
119	2.054	4	0.599	2.727	127.463	99	0.029	–
120	2.771	4	0.852	2.598	88.883	85	0.365	–
121	2.182	4	0.681	2.574	101.650	95	0.302	–
122	0.975	4	0.440	3.309	218.926	157	0.002	–
123	1.794	4	0.635	3.122	100.004	103	0.565	–
125	1.293	4	0.438	3.040	140.223	121	0.112	–
126	2.277	4	0.722	2.611	113.623	95	0.094	–
127	1.952	4	0.792	3.055	112.558	99	0.166	–
128	1.473	4	-0.365	3.216	103.207	94	0.242	ADS
129	2.220	5	0.515	2.683	138.572	119	0.106	OCDS
130	2.292	5	0.849	3.077	91.509	91	0.465	OCDS
131	1.890	5	0.355	3.667	138.402	122	0.147	OCDS
132	2.038	5	0.658	3.158	149.672	119	0.030	OCDS
133	2.176	5	0.436	2.881	137.927	136	0.438	OCDS
134	2.045	5	0.191	3.106	128.267	124	0.378	OCDS
135	1.870	5	0.364	3.820	131.215	112	0.104	OCDS
136	1.573	5	0.729	3.695	175.486	134	0.009	OCDS
137	2.210	5	0.039	3.130	114.340	96	0.098	OCDS
139	1.856	9	0.303	2.717	182.279	174	0.318	AAAQ
140	0.938	9	-0.617	2.793	243.842	266	0.831	AAAQ
141	1.669	9	0.128	2.676	235.426	199	0.039	AAAQ
143	2.016	9	0.384	2.746	182.436	168	0.211	AAAQ
144	1.249	9	0.263	2.746	234.435	192	0.020	AAAQ
145	1.835	9	0.544	2.969	146.549	150	0.564	AAAQ
146	1.838	9	0.328	2.604	215.826	180	0.035	AAAQ

### Construction of the Final Item Bank for the CAT-AUD

**FIGURE 1:**
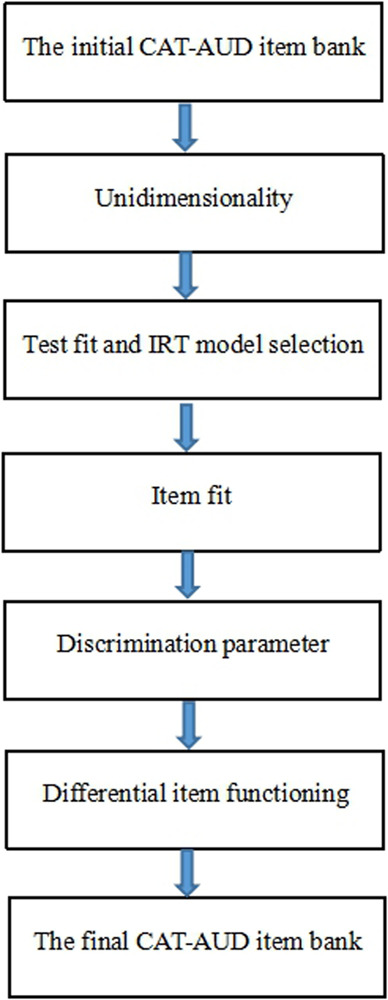
The steps of the psychometric measurement requirements of IRT used for the item bank generation.

To select high-quality items from the initial bank under the framework of IRT, unidimensionality test, item-fit test, discrimination test, and differential item functioning (DIF) test were performed in this study based on the initial item bank. Only those items meeting the requirements of IRT psychometric measurement can be involved in the final item bank to build the CAT-AUD. In order to understand the steps of item bank generation more intuitively, we provided a figure depicting the steps of the psychometric measurement requirements of IRT for the item bank generation (see Figure 1).

#### Unidimensionality

In IRT, unidimensionality implies that one major ability or trait should “explain” or “account” for the test performance of examinees (Hambleton and Swaminathan, 2013). The criteria for the data which might be reasonably well fit by a unidimensional model by exploratory factor analysis (EFA) included: (1) the ratio of the first eigenvalue to the second eigenvalue was above 3 (Lord, 1980; Hattie, 1985); (2) the percentage of variance interpreted by the first factor was more than 20% of the total variance (Reckase, 1979). Only two criteria were both satisfied at the same time, the data of the CAT-AUD item bank might fit the unidimensionality model well (Reckase, 1979). The software SPSS 21.0 was used for the analysis of unidimensionality.

#### Test Fit and IRT Model Selection

To assess the degree of fitness for the whole item bank, the graded response model (GRM; Samejima, 1969) and the generalized partial credit model (GPCM; Muraki, 1992) dealing with polytomous-scored items were used in this study. Given the three test-level model-fit indices: negative twice Log-likelihood (−2LL; Spiegelhalter et al., 1998), Akaike’s information criterion (AIC; Akaike, 1974), and Bayesian information criterion (BIC; Schwarz, 1978), the model representing the indices with the relatively smaller values were taken as the optimal model for the subsequent analysis.

#### Item Fit

In the test-fit test, the optimal model was selected and the overall degree of data fitting was suggested. However, this did not indicate that each item could fit the optimal model well. Accordingly, the degree of item-fit further should be explored. The S-χ^2^ was adopted to show the degree of the item-fit index (Orlando and Thissen, 2000, 2003). According to Flens et al. (2017), items with a p-value of S-χ^2^ below 0.001 were considered to be poor item-fit. A stricter standard was taken here, namely, if the item’s *p* < 0.01, it would be considered a low item-fit one and then deleted.

#### Discrimination Parameter

The high discrimination (slope) parameter of the IRT model refers to a desirable characteristic of the item quality (Masters, 1988). An item with relatively high discrimination is capable of ensuring that each subject’s latent trait level is separated by the effective area of the item bank. Given the study of Fliege et al. (2005), items whose discrimination values are <0.7 should be excluded from the item bank.

#### Differential Item Functioning

The DIF analysis was conducted to identify systematic errors attributed to different groups (Zumbo, 1999). If an item had DIF, the probability of selecting the same item type might be different for groups at the identical latent trait level. In current study, DIF for gender (male/female) and region (rural/non-rural) was investigated. The ordinal logistic regression (LR) (Crane et al., 2006) was used for the DIF analysis. The McFadden’s pseudo ΔR2 ≥ 0.2 (Flens et al., 2017) revealed a functional difference in the item, which should be removed. The R package lordif (Version 0.3–3; Choi et al., 2011) was used for the DIF test.

Through the above steps of the IRT analysis, the remaining items of the final item bank for the CAT-AUD would be employed to reassess the person ability parameter.

### CAT-AUD Simulation Study

The simulation study based on real participants’ response data was presented to investigate the characteristic, the criterion-related validity, as well as the predictive utility (sensitivity and specificity) of the CAT-AUD, AUDIT, and AUDIT-C for comparison. Before the investigation, the authors made some specifications about the program of the CAT-AUD.

#### Initial Item Selection

In the CAT-AUD simulation program, items which were administered to participants were based on their responses to prior items. At the beginning of the test, the initial item of the CAT-AUD was randomly selected from the final item bank since there was no response information of participants (Magis and Barrada, 2017).

#### Item Selection Algorithm

After the initial item selected, the Fisher information was picked as the selection index for the subsequent item selection progress (Baker, 1994). The item with the maximum Fisher information (MFI) based on the participant’s current theta in the rest item bank would be administered by subjects (Baker, 1994).

#### Scoring Method

Expected *a posteriori* method (EAP; Bock and Mislevy, 1982) is one of the Bayesian approaches to estimate the ability parameter. The prior distribution of ability is generally assumed to be a standard normal distribution. EAP is considered as an efficient estimation method of the person parameter. It can obtain the expected estimation of the person parameter by a direct calculation rather than an iteration.

#### Stopping Rule

In this study, the two commonly used CAT stopping rules (a fixed measurement of standard error and a fixed number of items) were integrated as the stopping rule of the CAT-AUD. The selection of standard error (SE) could ascertain the corresponding IRT reliability. As the subject answered a growing number of items, the measurement SE would be lower, and the reliability of the test would be higher. According to the existing studies (Wainer et al., 2000), when IRT reliability of r_xx_ ≥ 0.9, the result of the test could be considered credible and accurate. When the mean and standard deviation (SD) of theta are fixed to 0 and 1, the relationship between the IRT reliability and SE of the test is expressed as (Samejima, 1994):

$$r_{\mathrm{xx}}=1-S\,E\,{\left(\theta_{\text{i}}\right)}^{2}$$

According to the formula above, when the reliability reaches 0.9, the corresponding SE is around 0.32. If the reliability of each CAT procedure is expected to be at least 0.9, the corresponding maximum SE set to 0.32 will be relatively reasonable and acceptable. The maximum length of the test was set as 35, if the subject in testing has not reached the standard of SE ≤ 0.32 after answering 35 items, the CAT program would stop administering any items to the subject.

#### The Characteristics of the CAT-AUD

To explore the characteristics of the CAT-AUD, frequency distributions of theta values estimated via the full-length item bank and via the CAT-AUD were formed and compared first. Subsequently, the number of items administered by subjects, and the marginal reliability, representing the average of the corresponding reliability values under each theta (Smits et al., 2011), were investigated completely.

#### The Validity and Predictive Utility (Sensitivity and Specificity) of the CAT-AUD, AUDIT, and AUDIT-C

To investigate the validity of the CAT-AUD, the two scales including the AUDIT and AUDIT-C were introduced for the transverse comparison. The criterion-related validity of the CAT-AUD, AUDIT, and AUDIT-C was examined by assessing the Pearson’s correlation between individuals’ estimated theta values by these three tests and individuals’ corresponding raw scores of the MAST.

For the assessment of the predictive utility, the area under (AUC) the receiver operating characteristic curve (ROC) was introduced (Smits et al., 2011). The AUC refers to the probability that a randomly chosen patient is rated or ranked as more likely to be diseased than a randomly chosen healthy individual (Hajian-Tilaki, 2013). Youden Index (YI; Schisterman et al., 2005) is another major summary statistic of the ROC used for the interpretation and assessment, defining the maximum potential effectiveness of a test. YI equals the sum of the sensitivity and the specificity minus 1. The sensitivity (Se) refers to the probability that an AUD high-risk individual will be diagnosed with the disease, and the specificity (Sp) represents the probability that a healthy individual will be diagnosed with non-disease. The larger the value of these two indicators (Se and Sp), the better the diagnostic effect will be. The cut-off point is used to determine whether the estimate results show a clinical risk of subjects. In CAT-AUD, if the subjects’ theta value is higher than the cut-off point, they will be at risk of AUD; if not, they will be relatively healthy. At the cut-off point, the corresponding value of YI is the largest among all subjects’ tests. The MAST scale acted as a categorical variable of AUD and theta values estimated by the CAT-AUD, AUDIT, and AUDIT-C as the continuous variable. Subsequently, the ROC result was analyzed and portrayed using SPSS 21.0 software when the stopping rule was SE ≤ 0.32.

## Results

### Construction of the Item Bank for the CAT-AUD

#### Unidimensionality

According to the EFA results of the initial item bank, there were 5 items with factor loadings on the first factor <0.3 (Nunally, 1978). After these items were excluded, the unidimensionality test for the rest items was performed. The eigenvalue of the first component and the second component were 53.55 and 4.83, respectively. Their ratio of the first component and the second component was 11.09, which was far more than the criteria of 3 (Lord, 1980; Hattie, 1985). Moreover, the first factor interpreted about 41.84% of all the variance, which was more than the criteria of 20% (Reckase, 1979). The above results indicated the remained items could fit the unideminsional model well. And the ability parameters estimated based on the data were related to the first principal component.

#### Test Fit and IRT Model Selection

The commonly used test fit indicators (−2LL, AIC, BIC) of the GRM and the GPCM are listed in Table 3. It was found that the fitting degree of the GRM was better than that of the GPCM. Based on such results, the GRM was subsequently selected as the IRT model with the current data for the subsequent analysis.

**TABLE 3:** Test-fit results for two polytomous-scored IRT models.

**Model**	**−2LL**	**AIC**	**BIC**
GRM	129596.5	130564.5	132896.9
GPCM	131514.16	132482.2	133788.8

*GRM, Graded Response Model; GPCM, Generalized Partial Credit Model; −2LL, Negative twice Log-Likelihood; AIC, Akaike’s information criterion; BIC, Bayesian information criterion.*

#### Item-Fit, Discrimination and Differential Item Functioning

The result of item-fit suggested that item 65 and item 94 did not fit the GRM well. For discrimination parameters, there were 3 items (138, 142, 147) <0.7. In terms of DIF, there was no DIF item in area groups or gender groups.

After 10 items that did not meet the above requirements of psychometric measurement from the initial item bank were deleted, the final item bank remained 113 items. The number of items in the item bank measuring each symptom criteria of AUD defined in DSM-5 varied from 6 to 15 with an average of 11.27 (*SD* = 3.10), which indicated that the questionnaire based on the AUD item bank could exhaustively measure each diagnose criterion for AUD in DSM-5. And when the item bank was used to assemble the CAT-AUD, the probability that the CAT-AUD would have adequate content validity was high (Waltz et al., 2005).

The item psychometric information of the final item bank is listed in Table 2. As shown in Table 2, the slope value ranged from 0.93 to 4.07 with an average of 2.34 (*SD* = 0.80), suggesting that the quality of the final item bank was relatively high. The threshold of the final item bank ranged from −0.62 to 3.82, indicating that the item bank’s coverage of the theta value is skewed toward the high-risk group.

In Table 4, the descriptive statistics for the full-length item bank score distribution were shown. Evidently, the number of subjects with scores ranging from 0 to 49 is the largest, followed by those with scores ranging from 50 to 99, and those with the least number of subjects ranging from 250 to 299. In general, the standard deviation of each score range is close to each other. The standard deviation is the lowest for a sample whose score range is 200–249, and the standard deviation is the highest for a sample whose score range is 50–99.

**TABLE 4:** The descriptive statistics for the full-length item bank score distribution.

**Score range**	**The number of sample**	**Mean**	***SD***
0–49	489	25.79	13.094
50–99	229	70.10	13.661
100–149	114	119.40	13.752
150–199	43	169.91	14.635
200–249	29	222.66	12.743
250–299	11	264.09	13.575

### The CAT-AUD Simulation Study

#### The Characteristics of the CAT-AUD

**FIGURE 2:**
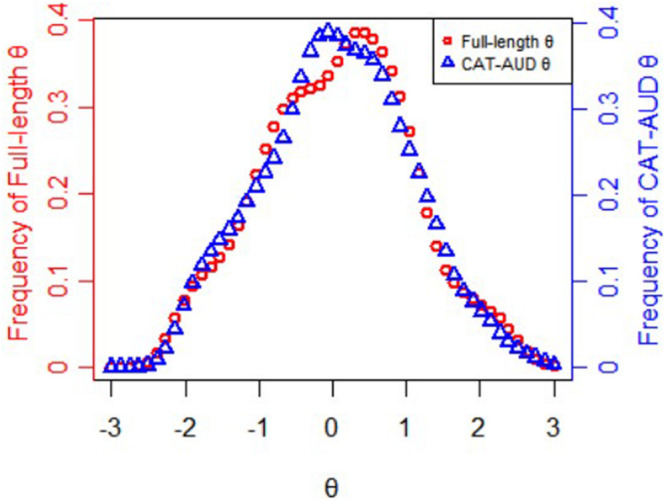
Frequency distributions of theta values estimated via the full-length item bank and the CAT-AUD at a given SE level (SE ≤ 0.32).

Figure 2 displays frequency distributions of theta values estimated via the full-length item bank and via the CAT-AUD at a given SE level (SE ≤ 0.32). The theta values(*M* = 0.06, *SD* = 1.002) estimated via the full-length item bank and the theta values(*M* = 0.05, *SD* = 0.987) estimated via the CAT-AUD were almost matched on frequency distributions, with a Pearson’s correlation of 0.95 (*p* < 0.01).

**FIGURE 3:**
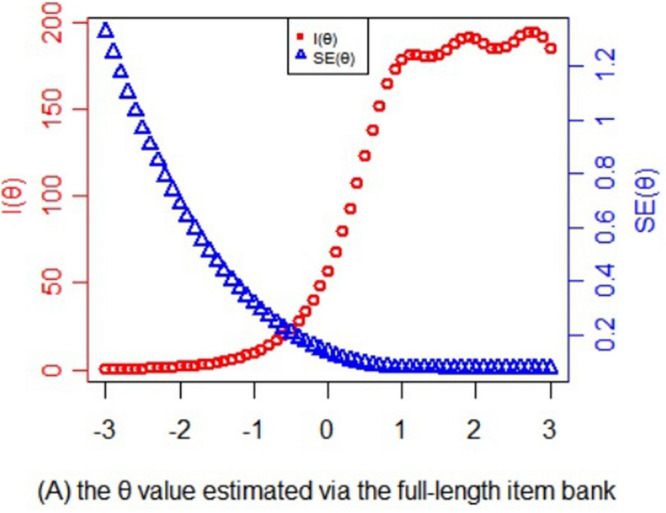
Statistical information and standard error function of the theta values estimated via the full-length item bank.

Figure 3 presents the information and its corresponding SE function of theta values estimated by the full-length item bank. Obviously, for those whose theta values are <−2, the information provided by the item bank is limited that it is almost impossible to provide accurate test results. For the subjects whose theta values ranged from −2 to −0.8, the test could provide certain amount of information. For the subject whose theta value is more than −0.8, the test could provide large amount of information and high reliability. With the increase of the theta value, the amount of information for item bank increases rapidly. When the theta value reaches about 1.5, the corresponding SE of the full-length test approaches 0. Therefore, for high-risk and clinical subjects, the item bank is very suitable and accurate for them, while for non-clinical subjects, the item bank can provide very little information.

**FIGURE 4:**
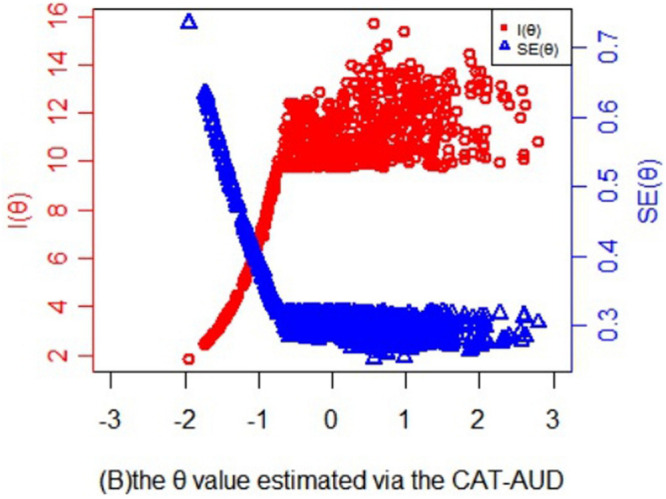
Statistical information and standard error function of the theta values estimated via the CAT-AUD at a given SE level (SE ≤ 0.32).

In Figure 4, the information and its corresponding SE function of theta values estimated via the CAT-AUD was shown. As can be seen, when the SE value is <0.32, theta value in the graph would be roughly >−1. The subjects whose theta values are >−1 could benefit a lot from the CAT-AUD as the amount of information are relatively high. While for the subjects whose theta values are <−1, the current CAT may not provide much information for them.

**FIGURE 5:**
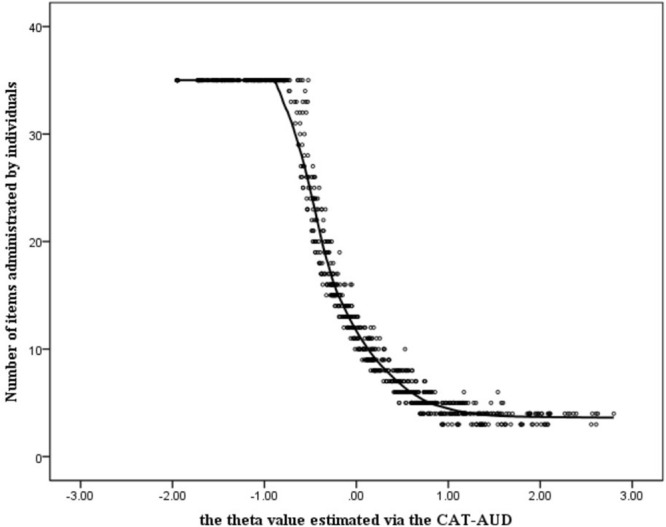
Number of items administered by individuals in the CAT-AUD at a given SE level (SE ≤ 0.32).

In Figure 5, the minimum number of items to be administrated by participants was around 3. And the average number of items to be answered was 16.00 (*SD* = 12.26), which only took up 14.16% of the item bank length. For individuals whose theta values of the CAT-AUD were <−0.5, the number of item usage remained unchanged and were all set at a maximum of 35 items. For those with a value >−0.5, the number of item usage decreased as theta increased until the theta value reached around 1. Moreover, the marginal reliability of the CAT-AUD (SE ≤ 0.32) was 0.87, indicating that the CAT-AUD had high reliability in estimating the person parameter.

#### The Validity and Predictive Utility (Sensitivity and Specificity) of the CAT-AUD, AUDIT, and AUDIT-C

Table 5 reveals the criterion-related validity between the criterion scale (MAST) and three questionnaires (CAT-AUD, AUDIT, AUDIT-C). As can be seen from the result, the criterion-related validity between the MAST and the CAT-AUD was the highest among three criterion-related validity values, which is suggesting that the estimated theta by the CAT-AUD was consistent with the result of the MAST.

**TABLE 5:** The predictive utility (sensitivity and specificity) of the CAT-AUD, AUDIT, and AUDIT-C and the criterion-related validity.

**Questionnaire**	**AUC(95%CI)**	**Cut-off**	**Se**	**Sp**	**YI**	**Criterion-related validity**
CAT-AUD	0.856 (0.830–0.881)	0.384	0.751	0.820	0.571	0.653
AUDIT	0.817 (0.787–0.848)	0.354	0.709	0.841	0.550	0.582
AUDIT-C	0.691 (0.656–0.727)	0.330	0.475	0.820	0.295	0.370

For the predictive utility, the performance of the CAT-AUD remains the best among the three questionnaires. To be specific, the AUC value of the CAT-AUD in Table 5 equaled to 0.856, which was higher than those of the AUDIT and AUDIT-C. The situation that the cut-off value of the CAT-AUD was higher than that of the AUDIT and AUDIT-C mirrors that the CAT-AUD could distinguish AUD high-risk individuals with higher severity in AUD than the other two questionnaires. The sensitivity of the CAT-AUD was the highest while the specificity of AUDIT was the highest.

## Discussion

In the current research, the CAT-AUD was developed to screen the AUD high-risk group and measure individuals’ severity of AUD. For these ends, a high-quality item bank was established for the CAT-AUD under the guidance of IRT to support the operation of the CAT-AUD simulation study.

The result of the study suggested that the CAT-AUD could provide accurate, valid psychometric information for the AUD high-risk group while it can only provide the healthy group with a low amount of information. From the perspective of screening high-risk group, the CAT-AUD achieved its intended goal. Specifically, the full-length item bank questionnaire and the CAT-AUD could provide accurate results for the AUD high-risk group and provide relatively inaccurate results for the healthy group. For the AUD high-risk group, the questionnaire could provide much more information than the healthy group. For instance, when the theta value was >−0.8, the corresponding SE was about <0.32 in Figure 3. When the mean and SD of theta values are fixed to 0 and 1 (Samejima, 1994), according to the relationship of reliability and SE, the corresponding reliability was above 0.9, which confirms that the CAT-AUD prefers to the AUD high-risk group. This parallels to the research in which items did not appear capable of precisely measuring subjects with low transmissible liability index (TLI) values (Kirisci et al., 2012). Because the fixed-length and the fixed SE roles were set in advance, the CAT-AUD could provide the reliability of about 0.9 in each test for participants. Hence, the bias problem which happened in the full-length questionnaire was attenuated but not completely avoided in the CAT-AUD. This situation indicates that the CAT-AUD was almost equivalent to the full-length questionnaire in terms of the test accuracy. As for the test efficiency, the performance of the CAT-AUD was far more different from the full-length questionnaire. Participants who took part in the full-length questionnaire were requested to complete tedious 123 items. Nevertheless, unlike the full-length questionnaire, participants with various levels of AUD would just be provided a few or dozens of items that are suitable for their estimated theta level, and the test constructed by these items could excavate much valid IRT information for each individual. Accordingly, CAT cannot only provide accurate test results for AUD high-risk group but also greatly reduce the burden of all subjects (high-risk group and healthy group) in testing.

The advantages of the CAT-AUD could be also suggested from the perspective of validity. It can be obtained from Table 5 that the CAT-AUD was more consistent with the screening of the criterion scale, while the consistency of the AUDIT and AUDIT-C was weaker. Besides, the CAT-AUD also showed a higher predictive utility than the other two questionnaires. The AUC value was higher than that of the AUDIT and AUDIT-C, and its sensitivity is the highest among the three questionnaires. Accordingly, though the 10-item AUDIT and the 3-item AUDIT-C can noticeably save the test time for individuals, the test validity is not as good as that of the CAT-AUD. The CAT-AUD can narrow the length of the test based on ensuring the accuracy of the test, and the average number of items used is only 16. Therefore, it can be considered that the CAT-AUD can serve as a reliable and efficient test tool for research and screening.

Though the results of the CAT-AUD are promising, some limitations remained. First, the valid subjects from all over the country in this study were 915, which is only a moderate size sample. When the sample size was enlarged, the research conclusion of AUD might be modified. So the influence of sample size on test results remains to be further studied. Second, since the CAT-AUD cannot provide sufficient information for those participants with a low AUD level, items with different levels of threshold (especially those with low levels of a threshold) should be added. Only when the item bank covers the whole range of theta value, can the CAT program provide more suitable items for the subjects with different theta values and also assess the potential trait level of the subjects more accurately. Third, in a real scenario, the response of participants will be affected by numerous factors in that the simulation research with real data may differ from the real experiment in many aspects (Smits et al., 2011). According to the results of existing researches (Kocalevent et al., 2009), the performance of CAT simulation was consistent with real administration results, but it is uncertain that the same conclusion could be generalized to real working CAT-AUD system. Hence, the real CAT-AUD administration is worth applying in real life to explore the efficiency, accuracy and other characteristics of the CAT-AUD. Fourth, in the current study, a scale proven to have good reliability and validity rather than a professional clinical diagnosis from health care institutions was selected as the criterion in that way the AUD high-risk individual rather than the AUD patients would be screened out. As a consequence, the result of the CAT-AUD for individuals is highly correlated with the diagnostic result. Furthermore, though the item bank of this study complies with the standard of unidimensionality, it is a chance to consider conducting the above studies under the framework of Multi-dimensional IRT (MIRT) to report symptoms information of AUD defined in DSM-5, which may be a very noteworthy and exciting entry point for subsequent studies.

## Data Availability Statement

The datasets generated for this study are available on request to the first author.

## Ethics Statement

The studies involving human participants were reviewed and approved by the Research Center of Mental Health, Jiangxi Normal University. The patients/participants provided their written informed consent to participate in this study. Written informed consent was obtained from the individual(s) for the publication of any potentially identifiable images or data included in this manuscript.

## Author Contributions

JL was responsible for the data processing and manuscript writing. DT and YC guided the process of the data processing and the manuscript writing. CX was responsible for the data collection. All authors contributed to the article and approved the submitted version.

## Conflict of Interest

The authors declare that the research was conducted in the absence of any commercial or financial relationships that could be construed as a potential conflict of interest.
